# Switching from a traditional undergraduate programme in (clinical) pharmacology and therapeutics to a problem-based learning programme

**DOI:** 10.1007/s00228-020-03027-3

**Published:** 2020-10-23

**Authors:** David J. Brinkman, Teresa Monteiro, Emilia C. Monteiro, Milan C. Richir, Michiel A. van Agtmael, Jelle Tichelaar

**Affiliations:** 1 Section Pharmacotherapy, Department of Internal Medicine, Amsterdam University Medical Centers, De Boelelaan 1117, 1081 HV Amsterdam, The Netherlands; 2 Research and Expertise Center in Pharmacotherapy Education (RECIPE), Amsterdam, The Netherlands; 3 Faculdade de Ciências Médicas, NOVA Medical School, Universidade NOVA de Lisboa, Lisbon, Portugal

**Keywords:** Clinical pharmacology, Therapeutics, Prescribing, Students, Undergraduate, Medical curriculum

## Abstract

**Purpose:**

The pharmacology and clinical pharmacology and therapeutics (CPT) education during the undergraduate medical curriculum of NOVA Medical School, Lisbon, Portugal, was changed from a traditional programme (i.e. discipline-based, lectures) to a problem-based learning (PBL) programme (i.e. integrated, case-based discussions) without an increase in teaching hours. The aim of this study was to investigate whether this change improved the prescribing competencies of final-year medical students.

**Methods:**

Final-year students from both programmes (2015 and 2019) were invited to complete a validated prescribing assessment and questionnaire. The assessment comprised 24 multiple-choice questions in three subdomains (working mechanism, side-effects and interactions/contraindications), and five clinical case scenarios of common diseases. The questionnaire focused on self-reported prescribing confidence, preparedness for future prescribing task and education received.

**Results:**

In total, 36 (22%) final-year medical students from the traditional programme and 54 (23%) from the PBL programme participated. Overall, students in the PBL programme had significantly higher knowledge scores than students in the traditional programme (76% (SD 9) vs 67% (SD 15); *p* = 0.002). Additionally, students in the PBL programme made significantly fewer inappropriate therapy choices (*p* = 0.023) and fewer erroneous prescriptions than did students in the traditional programme (*p* = 0.27). Students in the PBL programme felt more confident in prescribing, felt better prepared for prescribing as junior doctor and completed more drug prescriptions during their medical training.

**Conclusion:**

Changing from a traditional programme to an integrated PBL programme in pharmacology and CPT during the undergraduate medical curriculum may improve the prescribing competencies of final-year students.

**Electronic supplementary material:**

The online version of this article (10.1007/s00228-020-03027-3) contains supplementary material, which is available to authorized users.

## Introduction

Medical graduates should be able to prescribe rationally (i.e. effectively, safely and at low cost), because after graduation they go directly into clinical practice and prescribe drugs on a daily basis, often with minimal supervision [1]. Poor prescribing may lead to prescribing errors and adverse drug events, which may cause prolonged hospital stays, unplanned hospital readmissions, significant morbidity and mortality and high healthcare costs [2, 3]. Unfortunately, a significant proportion of medical graduates seem not to have acquired sufficient knowledge, skills and attitudes required for rational prescribing [1, 4–6]. This might be because of inadequate clinical pharmacology and therapeutics (CPT) education during the undergraduate medical curriculum. Indeed, most medical curricula in European medical schools devote little time to CPT education, which is still mainly based on traditional teaching/learning methods (e.g. self-study, lectures and written examinations) [7, 8]. Compared with problem-based learning (PBL) methods, traditional methods seem to be associated with a lower level of prescribing knowledge and skills among medical students [1]. PBL is intended to simulate active learning and enables students to work together in groups and learn about a subject in the context of a real problem, such as case-based discussions [9]. Interest in PBL has increased over the last 30 years, and many PBL courses in CPT have been shown to increase the prescribing competence of medical students [10–25]. However, most of these studies evaluated the short-term effect of a single course and very few evaluated the effect of an entire PBL programme on students’ prescribing competence before they graduate.

In NOVA Medical School, Lisbon, Portugal, the pharmacology and CPT programme of the undergraduate medical curriculum underwent a major revision in 2011. The old discipline-based programme consisted of one course in basic pharmacology in the third year and one course in therapeutics in the fourth year (total of 144 teaching hours). The course in the third year was mainly based on traditional learning methods, such as lectures and written assessments, and that in the fourth year focused on learning about therapeutic guidelines and was managed by clinicians from various disciplines. In the new programme, pharmacology was integrated with pathophysiology, microbiology and neurosciences in the second and third years and a new course in CPT was introduced in the fifth year (total of 129 teaching hours). These courses are all based on the principles of PBL and focus on solving written patient cases in working groups under the supervision of clinical pharmacologists. The aim of this study was to determine whether these changes improved the prescribing competence of final-year medical students.

## Methods

### Study design and participants

The impact of a new programme in pharmacology and CPT was measured using an observational study with a pre/post-test design in two different cohorts of final-year medical students from NOVA Medical School. Differences between the old and the new programmes are shown in Table 1. The first group (historic control) included students who completed the traditional programme in 2015 and the second group (PBL group) included students who completed the new programme in 2019. Students were recruited by the local teacher during regular teaching sessions, by email and/or with announcements on electronic notice boards. Participation was voluntary, anonymous and without consequences to prevent test-driven learning prior to the assessment. All participants were asked to complete an online assessment and questionnaire just before graduation. The study was approved by the ethics committee of NOVA Medical School (process no. 64/2018/CEFCM). Informed consent was obtained from all participants prior to inclusion.

**Table 1:** Differences in learning programmes

Traditional learning programme	Problem-based learning programme
Discipline-based	Integrated
Before 2011	After 2011
Pharmacologyː 112 teaching hours CPTː 32 teaching hours	Pharmacologyː 99 teaching hours CPTː 30 teaching hours
Pharmacologyː ECTS 9 credits CPTː ECTS 3 credits	Pharmacologyː ECTS 12 credits CPTː ECTS 3 credits
Pharmacologyː one course in the third year CPTː one course in the fourth year	Pharmacologyː two courses in the second and third year CPTː one course in the fifth year
Teachersː various clinicians	Teachersː clinical pharmacologists
Teaching methodsː lectures	Teaching methodsː lectures and case-based working groups
Assessment methodsː MCQ exam	Assessment methodsː active participation in each teaching session, MCQ exam

*CPT* clinical pharmacology and therapeutics, *ECTS* European Credit Transfer System, *MCQ* multiple-choice question

### Study materials

To evaluate prescribing knowledge and skills, we used a previously validated Web-based assessment tool and questionnaire [1]. The tool consisted of 24 multiple-choice questions (MCQs, knowledge) and five clinical case scenarios (skills). The MCQs covered three drug topics (i.e. mechanisms of action, side-effects and interactions and contraindications) and focused on the CPT knowledge that every medical graduate should have obtained before graduation (Supplementary Material A). The scenarios focused on essential diseases that medical graduates should know how to treat (i.e. essential hypertension, community-acquired pneumonia, acute bronchitis, osteoarthritis, gastroesophageal reflux) were presented in the same format, and were of comparable complexity (Supplementary material A). For each scenario, the student could choose to prescribe a new drug (maximum of two per scenario), not to prescribe any drug and/or to adapt current medication. If the student chose to prescribe a new drug, he/she had to complete an electronic prescription form, including drug name, dose, dosage, route of administration and treatment duration.

The standardized questionnaire has been used in previous studies [1, 26] and asked questions about demographics, self-reported confidence in prescribing skills (WHO 6-step [27]), estimated number of drug prescriptions written during the undergraduate medical curriculum for training purposes, evaluation of CPT education received and perceived preparedness for prescribing.

### Data collection and scoring

The assessment and questionnaire were completed by the historic control group in September 2015 and by the PBL group in June 2019. The assessment and questionnaire had to be completed within 60 min in a computer room at a scheduled time under the supervision of a local teacher. Prior to the assessment, all students were informed about the study objectives and received instructions. They were not allowed to use references or to consult each other, or the supervisor. The assessment was formative so that the results did not influence students’ grades at the university. This was done to prevent test-driven learning prior to the assessment which could bias the outcomes.

All MCQs were scored as correct or incorrect. The case scenarios were scored within a month after the assessment according to a specifically designed scheme based on corresponding Portuguese and international guidelines applicable at the time (Table 2) [28–31]. A clinical pharmacologist with a medical background (D.B.) scored each treatment plan as being inappropriate, suboptimal or appropriate. Subsequently, the same person screened the drug prescriptions for prescribing errors, as classified by Dean et al. [32]. Errors found were categorized by type.

**Table 2:** Scoring categories for the treatment plans

Category	Description	Examples (pain management of osteoarthritis*)
Appropriate	A treatment plan was considered appropriate if it was complete, effective, safe and low cost according to (inter)national guidelines	Prescribing a NSAID and a proton pump inhibitor to a patient with a history of a peptic ulcer and who already uses acetaminophen in maximum dosage
Suboptimal	A treatment plan was considered suboptimal if it was just below the standard of appropriate (e.g. the dose of the drug is slightly too high, less recommended drug choice)	Prescribing codeine to a patient with a history of a peptic ulcer and who already uses acetaminophen in maximum dosage
Inappropriate	A treatment plan was considered inappropriate if it was significantly below the standard of appropriate (e.g. potentially harmful drug interaction, relevant contraindication)	Prescribing a NSAID without a proton pump inhibitor to a patient with a history of a peptic ulcer and who already uses acetaminophen in maximum dosage

*Supplementary materials

### Data analysis

Descriptive variables are expressed as percentages with associated ranges. The two groups were compared using Mann-Whitney for continuous data and a chi-square test for categorical data. The Spearman correlation coefficient (*r*_s_) was used to analyse whether the number of drugs prescribed and self-rated confidence in prescribing was associated with skill scores. Knowledge scores were calculated as percentages of the maximum score. Data were collected in Excel format and analysed using IBM SPSS version 22.0 (IBM, Armonk, NY, USA). A *p* value of < 0.05 was considered significant.

## Results

In total, 36 (22%) final-year medical students from the traditional programme and 54 (23%) final-year medical students from the PBL programme participated. Table 3 shows the characteristics of both groups. Students in the new programme were significantly older than students in the traditional programme (median 24 versus 23 years, *p* = 0.002).

**Table 3:** Students’ characteristics

	Traditional programme *n* = 36	PBL programme *n* = 54	*P* value
Age (median, range)	23 (22–34)	24 (23–41)	0.002^a^
Sex (female, %)	56%	72%	0.10^b^
Portuguese nationality (%)	100%	100%	
Year of start study (%)			
2008	3%	-	
2009	17%	-	
2010	80%	-	
2012	-	5%	
2013	-	93%	
2014	-	2%	

*PBL* problem-based learning

^a^ Mann-Whitney

^b^Chi-square

### Knowledge and skills

Overall, students in the PBL programme had significantly higher knowledge scores than students in the traditional programme (mean 76% (SD 9) vs 67% (SD 15); *p* = 0.002). Also, students in the PBL programme had a significantly better knowledge of ‘mechanisms of action’ (90% (SD 14) vs 81% (SD 17); *p* = 0.006) and ‘side-effects’ (90% (SD 12) vs 74% (SD 20); *p* < 0.001). ‘Interaction and contraindications’ was the only drug topic for which scores were not significantly different between the two groups (48% (SD 18) vs 45% (SD 22); *p* = 0.62). Overall, students in the PBL programme made significantly fewer inappropriate therapy choices (*p* = 0.023) and fewer erroneous prescriptions than students in the traditional programme (*p* = 0.27) (Table 4). Therapy choices of both groups that were assessed as being ‘potentially harmful’ and ‘potentially lethal’ are described in detail in Supplementary Material B. The most common prescribing errors among both groups were ‘incomplete/incorrect drug prescription’, ‘less effective drug choice’ and ‘overdosing’ (Table 4).

**Table 4:** Skills of students in the traditional programme (*n* = 36) and problem-based learning programme (*n* = 54)

	Traditional programme *N* (%)	PBL programme *N* (%)	*p* value
Therapy appropriateness			
Total number of treatment plans	169	270	
Median number of treatment plans per student (range)	5 (3–5)	5 (4–5)	
Appropriate^a^	32 (19)	50 (19)	
Suboptimal^a^	32 (19)	82 (30)	
Inappropriate^a^	105 (62)	138 (51)	0.023^b^
Not immediately harmful^c^	88 (84)	135 (99)	
Potentially harmful^c^	12 (11)	3 (1)	
Potentially lethal^c^	5 (5)	0	
Prescriptions			
Total number of drug prescriptions	197	382	
Median number of drug prescriptions per student (range)	7 (4–10)	7 (5–10)	
Total number of prescribing errors	260	453	
Number of drug prescriptions including errors	160 (81)	288 (75)	0.27^d^
Types of errors^e^			
Drug not indicated or inappropriate for indication	29 (11)	19 (4)	
Less effective drug choice	64 (25)	56 (13)	
Underdosing	20 (8)	56 (13)	
Overdosing	28 (11)	63 (14)	
Too short duration	0 (0)	20 (4)	
Too long duration	15 (6)	63 (14)	
Undefined duration	2 (1)	29 (6)	
Incorrect drug form	0 (0)	4 (1)	
Incomplete/incorrect drug prescription	93 (36)	124 (27)	
Protecting medication omitted	6 (2)	12 (3)	
Inappropriate abbreviation	0 (0)	0 (0)	
Therapeutic duplicity	0 (0)	2 (0)	
Drug group name	3 (1)	4 (1)	
Other	0 (0)	1 (0)	

*PBL* problem-based learning

^a^ Percent of total number of treatment plans

^b^ Chi-square test

^c^Percent of total number of inappropriate treatment plans

^d^ Mann-Whitney

^e^ Percent of the total number of prescribing errors

### Attitudes

Overall, students in the PBL programme felt significantly more confident in their prescribing skills than students in the traditional programme (*p* < 0.001; Table 5), especially regarding ‘choose a (drug) treatment’. The only skill in which the students in the PBL programme did not feel more confident was ‘verify the suitability of your drug treatment’. A larger proportion of students in the PBL programme felt adequately prepared for their prescribing responsibilities as a junior doctor compared with students in the traditional programme (21% vs 9%).

**Table 5:** Self-rated confidence (WHO 6-step) of students in the traditional programme (*n* = 36) and problem-based learning programme (*n* = 54)

WHO 6-step	Traditional programme(max 5; ± SD)	PBL programme(max 5; ± SD)	*p* value
Step 1: define indication	3.1 ± 0.8	3.4 ± 0.9	
Step 2: specify therapeutic objective	2.9 ± 0.9	3.4 ± 0.9	
Step 3a: specify standard treatment	2.7 ± 1.0	3.3 ± 0.8	
Step 3b: verify the suitability of your treatment	2.6 ± 1.0	2.4 ± 0.9	
Step 4a: choose a (drug) treatment	2.3 ± 0.8	3.0 ± 1.0	
Step 4b: choose the correct dose and interval	1.6 ± 0.7	2.1 ± 1.0	
Step 4c: calculate the correct drug dose	1.8 ± 0.8	2.4 ± 1.1	
Step 5a: write a drug prescription	2.5 ± 1.0	2.9 ± 0.9	
Step 5b: give information and instructions	2.6 ± 1.0	3.2 ± 0.8	
Step 6: determine monitoring parameters	2.6 ± 0.8	2.8 ± 0.8	
Total	**2.5 ± 0.5**	**2.9 ± 0.5**	*< 0.001*^*a*^

*PBL* problem-based learning

^a^ Mann-Whitney

Most students in both the traditional and PBL programmes thought that too little time was devoted to clinical pharmacology (76% and 74%, respectively) and therapeutics (88% and 87%, respectively). Similarly, a significant proportion of students in both the traditional and PBL programmes rated the clinical pharmacology (39% and 51%, respectively) and therapeutics education (55% and 64%, respectively) as poor or very poor. During undergraduate training, 49% of the students in the traditional programme did not complete any drug prescription as opposed to 23% of the students in the PBL programme.

### Associations

Prescribing confidence was not associated with the prescribing skills scores of students in either the traditional programme (*r* = 0.20) or the PBL programme (*r* = 0.09). Similarly, the number of drug prescriptions was not strongly correlated with the skills scores of students in either the traditional programme (*r* = 0.21) or the PBL programme (*r* = −0.01).

## Discussion

This study shows that changing how pharmacology and CPT is taught, going from a traditional teaching programme to an integrated PBL programme, may increase prescribing competencies of final-year medical students. This effect was present even with a 10% decrease in the number of teaching hours. Students in the PBL programme had significantly better prescribing knowledge and skills than did students in the traditional programme. Additionally, they felt more confident in prescribing, felt better prepared for prescribing as junior doctor and completed more drug prescriptions during their medical training. However, since this is an observational study, our results should be interpreted with caution. Possible confounders such as increased awareness for pharmacotherapy in general over the last years, and CPT education in particular, and the increasing use of decision support systems might have influenced the results of the students in the PBL group. Nevertheless, results are in concordance with previous studies investigating the effect of PBL courses on prescribing knowledge, skills and attitudes of medical students [10–25]. Similarly, we previously showed that a context-learning programme for preclinical medical students, which is a more extreme form of PBL, leads to better prescribing during clinical clerkships [33]. However, this is one of the few studies to evaluate the effect of an entire PBL programme on students’ prescribing competence before they graduate. Other studies mainly evaluated the short-term effect of a single PBL course in one study year. Similar to our previous study [1], we found an increase in knowledge among students in the PBL programme, even though PBL is often said to lead to less factual knowledge in the basic sciences, such as pharmacology [34]. This might be explained by the fact that PBL is superior to traditional teaching methods in terms of long-term knowledge retention and the application of knowledge [35].

Our results suggest that integrating PBL modules in CPT education throughout the medical curriculum is associated with an increase in students’ prescribing competence at the end of the medical curriculum. Interestingly, the total number of CPT teaching hours was 10% lower in the PBL programme than in the traditional programme (129 vs 144 h), which suggests that the content and context of teaching are probably more important than the number of teaching hours. Nevertheless, the integration of training sessions throughout the medical curriculum can be challenging for medical schools with few CPT teachers. In order to reduce the workload of teachers, the Education Working Group of the European Association for Clinical Pharmacology and Therapeutics (EACPT) recommends near-peer teaching and the use of online learning resources, such as E-learning and E-books [8].

Despite an improvement in skills, a large proportion (51%) of the treatment plans of students in the PBL programme were still inappropriate (although not immediately harmful), and many drug prescriptions contained errors. This might be because although students learned to solve written patient cases, they received little practical teaching in simulated and real-life practice settings, as evidenced by the finding that only 23% of the students in the PBL programme had written out a prescription before they graduated.

A large proportion of students in the PBL programme still felt that they were not adequately prepared for their prescribing role and were not satisfied about the quantity and quality of CPT education. To further improve pharmacology and CPT education, we have the following recommendations, which are in line with those of the EACPT [36]. First, it is important that CPT is a clear and visible programme integrated longitudinally throughout the entire medical curriculum, starting as early as possible. In most European medical schools, CPT is taught in one or two separate courses and still accounts for a relatively small proportion of the total study load of the entire curriculum (1%) [8]. Second, to further improve students’ skills, it is essential that they take part in role-playing sessions with simulated patients and write drug prescriptions for real patients during clinical clerkships, under supervision of an experienced clinician. Third, it is also important that learning outcomes are compatible with the learning environment and assessment activities throughout the medical curriculum (constructive alignment). For example, skills should not be assessed with MCQs but with Objective Structured Clinical Examinations (OSCE) and workplace-based assessments [8]. Fourth, a valid and reliable prescribing assessment at or near the end of the curriculum is necessary to demonstrate that medical graduates have achieved the necessary competence to prescribe rationally. Unlike Portugal, some European countries such as the UK and the Netherlands already implemented such an assessment at a national level [37, 38]. In the UK, the Prescribing Safety Assessment (PSA) has shown to be feasible with the majority of final-year medical students meeting the prespecified standard of prescribing competence [37, 38]. A new Erasmus+ project is currently underway to develop, test and implement a standardised assessment on safe prescribing (EUroPE+) during the undergraduate medical curriculum in the European Union (EU) [39].

Although there was some improvement, students in both groups had a poor knowledge of drug interactions and contraindications (< 50% score) and did not feel confident in verifying the suitability of their treatment choice, as has also been found in other studies [1, 26]. One could question whether it is necessary that students have this knowledge because most electronic prescribing systems alert doctors to potentially harmful drug combinations and contraindications. However, prescribing systems often provide a high volume of irrelevant drug safety alerts, which could lead to ‘alert fatigue’ [40]. Additionally, the alerts provided by electronic prescribing systems have to be interpreted in the context of the specific patient; some alerts may be applicable, or not applicable, at the individual level. For these reasons, medical students should not reply to heavily on these decision support systems but learn to check the suitability of their drug choice for the specific patient context and know relevant drug interactions and contraindications by heart.

The finding that self-rated prescribing confidence was not strongly associated with assessed skills has been reported previously [26] and indicates that medical students do not accurately assess their prescribing skills. Since both lack of confidence and overconfidence might be harmful for patients, educational programmes should allow for discussion of this lack of self-knowledge with medical students.

Our study should be interpreted in the light of some limitations. First, this was an observational study with a historic control rather than a randomized controlled trial; however, because of practical reasons, it was not possible to withhold the PBL programme from any group of students. Second, the response rate of the participating students was very low; therefore, findings cannot be generalizable to the entire cohort. Despite the low response rate, the knowledge and skills of participating students might have been overestimated because students who participate voluntary are probably more motivated than average. Indeed, a recent study showed that students who participated in a non-mandatory pharmacology sessions were more likely to pass the final course exam than those who did not [41]. Third, the fact that students were not allowed to use references during the assessment differs from clinical practice in which they are allowed to do so. This may have negatively influenced students’ competencies. However, in clinical practice, doctors do not always have time to refer to relevant prescribing guidelines. Prescribing in these or acute situations is a reason why doctors should have ready knowledge and a broad skills set for common clinical problems. Fourth, the study was conducted in one medical school, which limits the generalizability of our results to other faculties and countries. Fifth, the fact that the therapeutics plans were reviewed by only one assessor limits the reliability of our results. Lastly, because the assessment was performed in a controlled environment, it can be questioned whether the same results would be found in daily clinical practice with all its attendant distractions.

## Conclusion

Taking the above limitations into account, we conclude that switching from a traditional programme to an integrated PBL programme with fewer teaching hours to teach pharmacology and CPT may be associated with increase in prescribing competencies among final-year medical students. Since prescribing skills were still not satisfactory, more attention should be given to training and explicitly assessing these skills in a simulated or clinical setting. In the future, it would be interesting to study the effect of an undergraduate PBL programme in larger cohorts and on the prescribing competence of junior doctors.

## Electronic supplementary material

ESM 1 (PDF 276 kb).

ESM 2 (DOCX 19 kb).

## Data Availability

The data that support the findings of this study are available on request from the corresponding author.
