# Supplementary material for: Switching from a traditional undergraduate programme in (clinical) pharmacology and therapeutics to a problem-based learning programme
Source: Eur J Clin Pharmacol. 2020 Oct 23;77(3):421–9. doi: 10.1007/s00228-020-03027-3 (PMC7867513; doi:10.1007/s00228-020-03027-3)
Supplement: Supplementary file 1 — (PDF 276 kb). [file 228_2020_3027_MOESM1_ESM.pdf]

European study on essential prescribing competencies - NOVA Medical School

Introduction

Thank you for participating in this study. The study is conducted on behalf of the Education Working Group of the European Association for Clinical Pharmacology and Therapeutics (EACPT).

The primary goal of this study is to assess essential prescribing competence of final-year medical students in Europe. In addition, we survey students' opinion about the prescribing education they have received during their medical study and their self-confidence in prescribing.

The measurement consists of 3 sections and takes approximately 45 minutes to complete:
Section 1. Informed consent and demographical questions
Section 2. Assessment of prescribing knowledge and skills
Section 3. Questionnaire about self-confidence in prescribing and received prescribing education

To start, please continue to the next page.

European study on essential prescribing competencies - NOVA Medical School

Section 2. Assessment of prescribing knowledge and skills

In this section, you have to solve 5 written patient cases. Each case consists of a brief description of a patient with a common disease. Your job is to decide whether you want to write a drug prescription for the patient or not. If you do not want to prescribe a drug, please select the option “no drug treatment”. If you do want to prescribe a drug, you have to fill in the "drug name", "drug dose", "drug dosage", "duration" and "route of administration" for each drug (maximum of 2 drugs per patient; also see the example given below). You can also stop a drug that the patient is already using.

After each case, you will have to answer multiple choice questions about essential drugs relevant for that patient case (please select the most appropriate answer option).

Please do NOT use any references/resources during the assessment.

Example how to fill in the drug prescription

Drug name	Amoxicillin
Drug dose	500 mg
Drug dosage	three times a day
Duration	5 days
Route of administration	oral

European study on essential prescribing competencies - NOVA Medical School

Section 2. Case 1

Situation: you are a junior doctor working in a primary health care centre.

The following patient comes to see you.

Mr. A.
Age: 54
Medical history: none.
Current medication: none.
Allergy: none.
Habits: 15 cigarettes per day, no alcohol.

One month ago, Mr. A came to your clinic with a history of acid reflux. Besides a body mass index of 29 kg/m2, there were no abnormal findings on physical examination. You advised him to lose weight, quit smoking and avoid foods that induce reflux.

Today, Mr. A. comes back to your clinic because the acid reflux is getting worse. About half an hour after every big meal, he complains about regurgitation (“taste of acid in the throat”), dysphagia and a burning pain in the epigastric region. He tells you that he quit smoking and also tried to lose some weight.

He is still overweight with a body mass index of 28 kg/m2. No abnormal findings are found on further physical examination.

An upper gastrointestinal endoscopy shows gastroesophageal reflux disease (GERD), Grade A.

Your working diagnosis is: **gastroesophageal reflux disease**, not sufficiently responding to life style changes.

*Only relevant information about the patient is given above. You may think you need more information by further history taking, physical or other examinations. If this information is not mentioned, you may assume that the findings are not relevant.

1. If you want to prescribe a drug for this patient, please fill in:

Drug name	
Drug dose	
Drug dosage	
Duration	
Route of administration	

2. If you want to prescribe another drug for this patient, please fill in:

Drug name	
Drug dose	
Drug dosage	
Duration	
Route of administration	

Example how to fill in the drug prescription

Drug name	Amoxicillin
Drug dose	500 mg
Drug dosage	three times a day
Duration	5 days
Route of administration	oral

3. If you want to stop a drug, please fill in the drug name:

[ ]

4. If you do NOT want to prescribe any drug for this patient, please select the option below:

○ I don't want to write a drug prescription for this patient.

THE EUROPEAN ASSOCIATION OF CLINICAL PHARMACOLOGY AND THERAPEUTICS
LEARN MORE

Recipe
Research & Expertise Center In Pharmacotherapy Education

European study on essential prescribing competencies - NOVA Medical School

Section 2. Acid reflux

Please answer the multiple-choice-questions below.

1. The main working mechanism of proton-pump inhibitors (e.g. omeprazole) concerns

- ◯ reducing the gastric acid secretion
- ◯ neutralizing gastric acid in the stomach
- ◯ bactericidal effects on Helicobacter pylori
- ◯ creating a protective coating over ulcers
- ◯ blocking dopamine receptors in the gastrointestinal system

2. The first-line treatment of peptic ulcers caused by Helicobacter pylori infection is a combination of antibiotics with

- ◯ H2-antagonist (e.g. ranitidine)
- ◯ antacid (e.g. aluminium hydroxide)
- ◯ proton-pump inhibitor (e.g. omeprazole)
- ◯ antiemetic (e.g. metoclopramide)
- ◯ anti-inflammatory agent (e.g. prednisolone)

3. Which of the following drugs is the most likely to increase the risk of peptic ulcers?

- ◯ heparin sodium
- ◯ ibuprofen
- ◯ metoclopramide
- ◯ phenytoin
- ◯ paracetamol (acetaminophen)

4. How can proton-pump inhibitors primarily alter the absorption of other drugs?

- ◯ by increasing the gastric pH
- ◯ by decreasing the gastric pH
- ◯ by formation of insoluble complexes
- ◯ by reducing the intestinal motility
- ◯ by reducing bile salt formation

5. The effect of which of the following drugs can be decreased when combined with omeprazole?

- miconazol
- clopidogrel
- coumarin
- tetracycline
- acetylsalicylic acid

6. What is a common symptom when a patient suddenly stops with a proton-pump inhibitor?

- diarrhoea
- regurgitation
- skin rash
- joint pain
- anaphylaxis

European study on essential prescribing competencies - NOVA Medical School

Section 2. Case 2

Situation: you are a junior doctor working in a primary health care centre.

The following patient comes to see you.

Mrs. B.
Age: 69
Medical history:
- peptic ulcer, 5 years ago: eradication treatment for 2 weeks. Since then occasionally dyspepsia for which she uses aluminium hydroxyde gel.
- osteoarthritis of both knees, since 1 year: confirmed by X-ray.

Current medication:
- aluminium hydroxyde 5mL gel, four times a day.
- paracetamol (acetaminophen) 1g tablet, three times a day.

Allergy: none.
Habits: no smoking, no alcohol.

Today, Mrs. B. comes to your practice and tells you that she has progressive pain in both knees, not responding to paracetamol (acetaminophen). She tells you that the pain is increasingly worsening during walking, and that rest does not help anymore. In the morning when she gets up her knees are stiff and painful. Mrs. B doesn't want any operation of her knees.

On physical examination you find no deformity of the knee joints. There is some crepitus on joint movement of both knees. No abnormal findings were found by further physical examination.

Your working diagnosis is: **pain due to osteoarthritis of both knees**, not sufficiently responding to treatment with paracetamol.

*Only relevant information about the patient is given above. You may think you need more information by further history taking, physical or other examinations. If this information is not mentioned, you may assume that the findings are not relevant.

1. If you want to prescribe a drug for this patient, please fill in:

Drug name	
Drug dose	
Drug dosage	
Duration	
Route of administration	

2. If you want to prescribe another drug for this patient, please fill in:

Drug name	
Drug dose	
Drug dosage	
Duration	
Route of administration	

Example how to fill in the drug prescription

Drug name	Amoxicillin
Drug dose	500 mg
Drug dosage	three times a day
Duration	5 days
Route of administration	oral

3. If you want to stop a drug, please fill in the drug name:

4. If you do NOT want to prescribe a drug for this patient, please select the option below:

◯ I don't want to write a drug prescription for this patient.

European study on essential prescribing competencies - NOVA Medical School

Section 2. Pain management

Please answer the multiple-choice-questions below.

1. Which of the following drugs inhibits the prostaglandin synthesis?

- ◯ fluoxetine
- ◯ amitriptyline
- ◯ ibuprofen
- ◯ morphine
- ◯ codeine

2. Which of the following drugs is most likely to cause respiratory depression?

- ◯ amitriptyline
- ◯ morphine
- ◯ ibuprofen
- ◯ paracetamol (acetaminophen)
- ◯ acetylsalicylic acid

3. Which of the following drugs is most likely to decrease the kidney function (eGFR)?

- ◯ paracetamol (acetaminophen)
- ◯ morphine
- ◯ prednisolone
- ◯ ibuprofen
- ◯ amitriptyline

4. Which of the following drugs is most likely to cause fatal liver damage when overdosed?

- ◯ ibuprofen
- ◯ codeine
- ◯ paracetamol (acetaminophen)
- ◯ amitriptyline
- ◯ acetylsalicylic acid

5. Which of the following drugs is most likely to enhance the risk of gastrointestinal bleeding conferred by NSAIDs?

- enalapril
- furosemide
- simvastatin
- fluoxetine
- amlodipine

6. Which of the following drugs should be prophylactically combined with oral laxative when prescribed?

- ibuprofen
- amitriptyline
- paracetamol (acetaminophen)
- morphine
- acetylsalicylic acid

European study on essential prescribing competencies - NOVA Medical School

Section 2. Case 3

Situation: you are a junior doctor working in a primary health care centre.

The following patient comes to see you.

Mr C.
Age: 35
Medical history:
- acute appendicitis, 23 years ago: appendectomy.
Current medication: none.
Allergy: cats.
Habits: no smoking, no alcohol.

Mr. C comes to your clinic because he doesn't feel well and has been coughing a lot recently. Since this morning, he has a temperature of 37.8 °C. He coughs up grey sputum. Besides some hoarseness and muscle pain, he has no other complaints.

On physical examination you find some wheezing and rhonchi over both lungs. No abnormal findings were found by further physical examination.

Your working diagnosis is: **uncomplicated acute bronchitis.**

*Only relevant information about the patient is given above. You may think you need more information by further history taking, physical or other examinations. If this information is not mentioned, you may assume that the findings are not relevant.

1. If you want to prescribe a drug for this patient, please fill in:

Drug name	
Drug dose	
Drug dosage	
Duration	
Route of administration	

2. If you want to prescribe another drug for this patient, please fill in:

Drug name	
Drug dose	
Drug dosage	
Duration	
Route of administration	

Example how to fill in the drug prescription

Drug name	Amoxicillin
Drug dose	500 mg
Drug dosage	three times a day
Duration	5 days
Route of administration	oral

3. If you want to stop a drug, please fill in the drug name:

4. If you do NOT want to prescribe a drug for this patient, please select the option below:

○ I don't want to write a drug prescription for this patient.

European study on essential prescribing competencies - NOVA Medical School

Section 2. Case 4

Situation: you are a junior doctor working in a primary health care centre.

The following patient comes to see you.

Mrs. D
Age: 66
Medical history:
- migraine
- essential hypertension, since 15 years.
- recurrent urinary tract infection, since 6 years.

Current medication:
- ibuprofen 200mg tablet, when needed.
- metoclopramide 10mg supp, when needed.
- amlodipine 5mg tablet, once a day.
- nitrofurantoin 100mg tablet, once a day.

Allergy: none.
Habits: stopped 5 years ago; 20 packyears, no alcohol.

Mrs. D comes to your practice because she is increasingly breathless. She has been coughing a lot over the last couple of days and feels sick. She coughs up yellow/green sputum. Since yesterday, her temperature is 40.0 °C.

On physical examination you find loud wheezing and low-pitch crackles on the right side of the chest. No abnormal findings were found by further history and physical examination.

The X-ray shows a consolidation in the lower lobe of the right lung.

Your working diagnosis is: **mild community-acquired pneumonia** (Pneumonia Severity Index: Class I, CURB-65 score = 1).

1. If you want to prescribe a drug for this patient, please fill in:

Drug name	
Drug dose	
Drug dosage	
Duration	
Route of administration	

2. If you want to prescribe another drug for this patient, please fill in:

Drug name	
Drug dose	
Drug dosage	
Duration	
Route of administration	

Example how to fill in the drug prescription

Drug name	Amoxicillin
Drug dose	500 mg
Drug dosage	three times a day
Duration	5 days
Route of administration	oral

3. If you want to stop a drug, please fill in the drug name:

4. If you do NOT want to prescribe a drug for this patient, please select the option below:

○ I don't want to write a drug prescription for this patient.

European study on essential prescribing competencies - NOVA Medical School

Section 2. Antibiotics

Please answer the multiple-choice-questions below.

1. Erythromycin belongs to which group of antibiotics?

- β-lactams
- macrolides
- tetracyclines
- aminoglycosides
- quinolones

2. Which pathway in bacteria is mainly inhibited by penicillin (e.g. amoxicillin)?

- RNA synthesis
- cell wall synthesis
- protein synthesis
- DNA synthesis
- folic acid metabolism

3. Which one of the following is a common early side effect of penicillin?

- constipation
- vision loss
- orthostatic hypotension
- skin rash
- tooth discoloration

4. Which of the following types of antibiotics is most likely to cause nephrotoxicity?

- penicillin
- macrolide
- aminoglycoside
- tetracycline
- quinolone

5. The absorption of tetracyclines (e.g. doxycycline) is reduced when taken with which of the following product(s)?

- ◯ tyramine-containing food (e.g. red wine, aged cheeses)
- ◯ grapefruit
- ◯ alcohol
- ◯ dairy products (e.g. milk, yogurt)
- ◯ high-fiber foods (e.g. whole grain)

6. Which of the following antibiotics should NOT be administered to children because of its potential for disrupting bone and tooth development?

- ◯ amoxicillin
- ◯ ciprofloxacin
- ◯ doxycycline
- ◯ ceftriaxone
- ◯ erythromycin

European study on essential prescribing competencies - NOVA Medical School

Section 2. Case 5

Situation: you are a junior doctor working in a primary health care centre.

The following patient comes to see you.

Mrs E.
Age: 47
Medical history:
- asthma bronchiale, since 25 years.
- atopic eczema, since 25 years.
- diabetes mellitus type II, since 7 years.
- hypercholesterolemia, since 7 years.
- essential hypertension, since ½ year.

Current medication:
- salbutamol 100µcg aerosol inhalation, when needed.
- beclomethasone 200µcg rotacaps inhalation, three times a day.
- hydrocortisone acetate cream 1%, twice a day to the affected area(s) of skin.
- metformin 850mg tablet, two times a day.
- simvastatin 10mg tablet, once a day.
- hydrochlorothiazide 12,5mg tablet, once a day

Allergy: none
Habits: 20 cigarettes per day, no alcohol.

Half a year ago, Mrs. E. had a blood pressure of 159/100 mmHg during a routine check. She had no complaints and no abnormal findings were found on physical examination. You told Mrs. E. to follow a diet with a low sodium and low cholesterol content, and advised her to take more physical exercise.

Three months ago, her blood pressure was 168/110 mmHg although she did follow your advice. She still had no complaints, and all other findings were normal. You wrote a drug prescription with hydrochlorothiazide 12,5mg, once a day.

Today, her bloodpressure is 157/98 mmHg. Again, all other findings are normal.

Your working diagnosis is: **essential hypertension**, not sufficiently responding to hydrochlorothiazide 12,5mg daily and life style changes.

*Only relevant information about the patient is given above. You may think you need more information by further history taking, physical or other examinations. If this information is not mentioned, you may assume that the findings are not relevant.

1. If you want to prescribe a drug for this patient, please fill in:

Drug name	
Drug dose	
Drug dosage	
Duration	
Route of administration	

2. If you want to prescribe another drug for this patient, please fill in:

Drug name	
Drug dose	
Drug dosage	
Duration	
Route of administration	

Example how to fill in the drug prescription

Drug name	Amoxicillin
Drug dose	500 mg
Drug dosage	three times a day
Duration	5 days
Route of administration	oral

3. If you want to stop a drug, please fill in the drug name:

4. If you do NOT want to prescribe a drug for this patient, please select the option below:

○ I don't want to write a drug prescription for this patient.

European study on essential prescribing competencies - NOVA Medical School

Section 2. Cardiovascular risk management

Please answer the multiple choice questions below.

1. Which of the following antihypertensive drugs is first choice treatment for a diabetes type II patient with microalbuminuria?

- ◯ amlodipine
- ◯ methyldopa
- ◯ hydrochlorothiazide
- ◯ enalapril
- ◯ bisoprolol

2. Which of the following antihypertensive drugs inhibits the reabsorption of sodium and chloride in the distal convoluted tubule of the nephron?

- ◯ enalapril
- ◯ furosemide
- ◯ amlodipine
- ◯ metoprolol
- ◯ hydrochlorothiazide

3. Which of the following types of antihypertensive drugs is most likely to cause hypokalemia?

- ◯ calcium antagonists
- ◯ beta-blockers
- ◯ ACE-inhibitors
- ◯ thiazide diuretics
- ◯ angiotensin receptor blockers

4. Which of the following antihypertensive drugs is most likely to cause bradycardia?

- ◯ enalapril
- ◯ hydrochlorothiazide
- ◯ metoprolol
- ◯ amlodipine
- ◯ spironolactone

5. Which of the following types of antihypertensive drugs can mask the symptoms of hypoglycemia in diabetes patients?

- ◯ calcium antagonists
- ◯ beta-blockers
- ◯ ACE-inhibitors
- ◯ thiazide diuretics
- ◯ angiotensin receptor blockers

6. What is a common result when an ACE-inhibitor is combined with NSAID?

- ◯ hyperkalemia
- ◯ hypokalemia
- ◯ hypernatremia
- ◯ hypocalcaemia
- ◯ hyperthermia

THE EUROPEAN ASSOCIATION OF CLINICAL PHARMACOLOGY AND THERAPEUTICS
LEARN MORE

Recipe
Research & Expertise Center In Pharmacotherapy Education

European study on essential prescribing competencies - NOVA Medical School

Section 3. Questionnaire about self-confidence in prescribing

How confident would you feel in the following skills when prescribing a drug by yourself?

1. Taking all the relevant information of the patient into consideration in prescribing

○ Very unconfident ○ Unconfident ○ Neutral ○ Confident ○ Very Confident

2. Specifying a specific, measurable and achievable therapeutic objective

○ Very unconfident ○ Unconfident ○ Neutral ○ Confident ○ Very Confident

3. Specifying a standard treatment (P-drugs) for a given diagnosis

○ Very unconfident ○ Unconfident ○ Neutral ○ Confident ○ Very Confident

4. Verifying the suitability of the treatment for the patient (e.g. checking all relevant contra-indications and interactions)

○ Very unconfident ○ Unconfident ○ Neutral ○ Confident ○ Very Confident

5. Choosing the correct (drug) treatment (i.e. effectively, safely, and at low cost)

○ Very unconfident ○ Unconfident ○ Neutral ○ Confident ○ Very Confident

6. Choosing the correct dose and interval of administration

○ Very unconfident ○ Unconfident ○ Neutral ○ Confident ○ Very Confident

7. Calculating the correct drug dose (e.g. for children)

○ Very unconfident ○ Unconfident ○ Neutral ○ Confident ○ Very Confident

8. Writing/typing a drug prescription

○ Very unconfident ○ Unconfident ○ Neutral ○ Confident ○ Very Confident

9. Giving the patient all relevant instructions, information and warnings of the prescribed drug

- ○ Very unconfident
- ○ Unconfident
- ○ Neutral
- ○ Confident
- ○ Very Confident

10. Determining all relevant monitoring parameters (e.g. plasma potassium, creatinine concentration)

- ○ Very unconfident
- ○ Unconfident
- ○ Neutral
- ○ Confident
- ○ Very Confident

THE EUROPEAN ASSOCIATION OF CLINICAL PHARMACOLOGY AND THERAPEUTICS
LEARN MORE

Recipe
Research & Expertise Center In Pharmacotherapy Education

European study on essential prescribing competencies - NOVA Medical School

Section 3. Questionnaire about prescribing education

1. How many hours of training in prescribing do you think you have received (so far) during your undergraduate study

Lectures and seminars

Workgroups and small-group sessions

Computer-based teaching and E-learning

Clinics (e.g. prescribing for real patients)

Individual teaching by supervisor (e.g. after having seen a patient)

Self-study

2. The number of times I wrote/typed a drug prescription during my undergraduate study (so far) is

Prescribing education can be divided into three disciplines, each with a specific focus:

1. Basic pharmacology: education about drug's pharmacological effect on the body (i.e. pharmacodynamics), and the action of drugs in the body over a period of time (i.e. pharmacokinetics).

2. Clinical pharmacology: education about the application of pharmacological principles and methods in the medical clinic and towards patient care and outcomes (e.g. drug interactions and contraindications).

3. Pharmacotherapy: education about the process of rational prescribing (i.e. how to choose a specific drug for a patient).

3. During my undergraduate medical study, I feel that the amount of teaching in

	Far too little	Too little	Just about right	Too much	Far too much
Basic pharmacology is					
Clinical pharmacology is					
Pharmacotherapy is					

4. During my undergraduate study, I rate the overall teaching of

	Very poor	Poor	Average	Good	Very good
Basic pharmacology	◯	◯	◯	◯	◯
Clinical pharmacology	◯	◯	◯	◯	◯
Pharmacotherapy	◯	◯	◯	◯	◯

5. During my undergraduate study, I would like more teaching in

	Yes	No	Unsure
Basic pharmacology	◯	◯	◯
Clinical pharmacology	◯	◯	◯
Pharmacotherapy	◯	◯	◯

6. I feel confident in how to find relevant, updated and unbiased information on drugs, as a support in the prescribing process

◯ Disagree ◯ Tend to Disagree ◯ Neutral ◯ Tend to Agree ◯ Agree

7. I feel that my medical curriculum prepared me adequately for my future prescribing responsibilities as a junior doctor

◯ Disagree ◯ Tend to Disagree ◯ Neutral ◯ Tend to Agree ◯ Agree

8. Would you like more experience in writing drug prescriptions for real patients before graduating?

◯ Yes ◯ No ◯ Unsure
