# Supplementary material for: Switching from a traditional undergraduate programme in (clinical) pharmacology and therapeutics to a problem-based learning programme
Source: Eur J Clin Pharmacol. 2020 Oct 23;77(3):421–9. doi: 10.1007/s00228-020-03027-3 (PMC7867513; doi:10.1007/s00228-020-03027-3)
Supplement: Supplementary file 2 — (DOCX 19 kb). [file 228_2020_3027_MOESM2_ESM.docx]

**Supplementary Material B.** Students’ therapy choices rated as being ‘potentially harmful (n= 15) and ‘potentially lethal’ (n= 5).

| Category | Traditional programme | PBL programme |
| --- | --- | --- |
| Potentially harmful  (n= 15) | *Osteoarthritis:*  Prednisolone 100 mg once a day orally for 5 days (drug inappropriate for indication with potential harmful side effects) | *Community-acquired pneumonia case:*  Amoxicillin 1000 mg together with clavulanic acid 200 mg intravenously twice a day for 7 days (incorrect drug form; intravenous therapy means that the patient has to be admitted to the hospital although there is no indication) |
|  | *Osteoarthritis:*  Prednisolone 40 mg once a day orally for one week (drug inappropriate for indication with potential harmful side effects) | *Essential hypertension case:*  Captopril 75 mg twice a day orally for three months (less recommended drug choice for indication and overdose) |
|  | *Osteoarthritis:*  Ibuprofen 1000 mg twice a day orally for undefined period together with omeprazole 400 mg once a day orally for undefined period (overdose with potential harmful side effects and incomplete prescription)  *Acute bronchitis case:*  Prednisone with unknown dosage, duration and route of administration (drug inappropriate for indication with potential harmful side effects and incomplete prescription) | *Gastroesophageal reflux case:*  Omeprazole 150 three items a day orally for one month (overdose with potential harmful side effects) |
|  |  |  |
|  | *Acute bronchitis case:*  Amoxicillin and clavulanic acid 500/200 mg three times a day orally for 6 days (drug inappropriate for indication with potential significant side effects) |  |
|  | *Acute bronchitis case:*  Amoxicillin and clavulanic acid with unknown dosage, duration and route of administration (drug inappropriate for indication with potential harmful side effects and incomplete drug prescription) |  |
|  | *Community-acquired pneumonia case:*  Levofloxacin 400 mg intravenously during three hours (less recommended drug and incorrect drug form; intravenous therapy means that the patient has to be admitted to the hospital although there is no indication) |  |
|  |  |  |
|  | *Gastroesophageal reflux case:*  Omeprazole 500 mg three times a day orally for 8 weeks (overdose with potential harmful side effects) |  |
|  | *Gastroesophageal reflux case:*  Omeprazole 100 mg twice a day orally for two weeks (overdose with potential significant side effects) |  |
|  | *Gastroesophageal reflux case:*  Omeprazole 200 mg twice a day orally for two weeks (overdose with potential harmfiul side effects) |  |
|  | *Gastroesophageal reflux case:*  Esomeprazole 400 mg twice a day orally for three weeks (overdose with potential harmful side effects) |  |
|  | *Gastroesophageal reflux case:* Omeprazole 200 mg once a day orally for one week (overdose with potential harmful side effects) |  |
| Potentially lethal (n= 5) | *Osteoarthritis:*  Morphine 300 mg once a day orally if needed (overdose with potential harmful side effects) |  |
|  | *Essential hypertension case:*  Enalapril 600 mg once a day orally for undefined period (drug inappropriate for indication with potential harmful side effects) |  |
|  | *Essential hypertension case:*  Enalapril 400 mg twice a day orally for one week until next consultation (overdose with potential harmful side effects) |  |
|  | *Essential hypertension case:*  Enalapril 100 mg twice a day orally for one month (overdose with potential harmful side effects)  *Essential hypertension case:*  Furosemide 500 mg twice a day orally for two weeks (drug inappropriate for indication with potential harmful side effects) |  |
|  |  |  |

PBL, problem-based learning.
